# Sleep disorders in cancer: interactions and intrinsic links

**DOI:** 10.3389/fonc.2025.1535442

**Published:** 2025-07-16

**Authors:** Chunxin Han, Nan Li, Xinlei Wang, Zhao Zhuang, Qianqian Cao, Shoushi Wang

**Affiliations:** Department of Anesthesia and Perioperative Medicine, Qingdao Central Hospital, University of Health and Rehabilitation Sciences (Qingdao Central Hospital), Qingdao, China

**Keywords:** cancer, sleep disorders, risk factor, treatment, mechanism

## Abstract

The continuous improvement of early cancer screening and treatment technologies can significantly increase the survival time of cancer patients. Though cancer gradually becomes a chronic disease that can be controlled through continuous treatment, the scientific community has begun to pay attention to the physical and emotional issues that cancer and its treatment cause for cancer survivors. This review focuses on sleep disorders, which is quite common yet frequently overlooked in cancer survivors. Epidemiological and experimental data have demonstrated an inherent relationship between cancer and sleep disorders. This relationship may harm treatment adherence, quality of life, and survival rate of patients. This article systematically introduces the epidemiology, influencing factors, and underlying mechanisms of cancer-related sleep disorders. The impact of sleep disorders on cancer prognosis and current common treatment measures is also discussed to enhance the understanding of healthcare workers in addressing cancer complications.

## 1 Introduction

Cancer ranks as the leading cause of mortality globally, second only to heart disease, and is the leading cause of death in Southeast Asia (1). Globally, there will likely be a 50% increase in new cases of cancer due to factors such as population growth, aging, lifestyle modifications, and advancements in early detection technologies. The International Agency for Cancer has released a statistical analysis stating that the number of cancer cases will increase from 18 million in 2018 to 27 million in 2040 (2). While the incidence of patient survival has increased due to ongoing advancements in medical technologies, this has also presented new difficulties for individuals and society. Many cancer survivors endure physical and mental health issues, typically secondary to cancer and its treatment. The common symptoms include pain, anxiety, depression, fatigue, sleep disorders, and cognitive impairment, and the frequency is two to three times higher than in the general population. In some situations, these symptoms may be linked to an increase in cancer-associated mortality, which could lower the quality of life of the survivor. Sleep disorders is a serious public health issue and has been shown to act as independent risk factors for mortality in patients with cancer, even when controlling for variables (3). Cancer and sleep interact and reinforce each other, through multiple mechanisms such as immunity, inflammatory response, neuroendocrine pathways, and DNA damage and repair. In recent years, sleep disorders in cancer survivors has received increasing attention from research scholars.

To identify studies exploring potential links between sleep disorders and cancer, we searched PubMed and Web of Science databases covering the last 10 years of studies published up to August 28, 2024, with no restrictions on study type or geographic boundaries to enhance the comprehensiveness of the literature search. The search strategy consisted of two themes, and search terms used for sleep disorders included “sleep” “sleep disorders” “insomnia” “sleep disruption” and “obstructive sleep apnea syndrome”. The following terms were used for cancer: “cancer” “malignant tumor” and “malignant tumour”. To avoid bias due to frequent database updates, all literature searches and data downloads were completed within 1 day, August 28, 2024.We manually screened observational studies using cross-sectional, case-control, retrospective, or prospective cohort designs, studies exploring the impact of sleep disorders on the development of cancer, and studies identifying sleep disorders in patients with cancer through questionnaires, interviews, or clinical diagnosis. This study provides a thorough introduction to the screening for and incidence of sleep disorders in patients with cancer, and analyzes the factors that may contribute to the onset of the symptom during cancer treatment, which will help in the prevention and treatment strategies for sleep disorders. In addition, the impact of sleep disorders on the body of patients with cancer, current common treatment measures, as well as the possible underlying mechanisms of sleep disorders and cancer are also discussed.

## 2 Screening

Sleep disorders generally refer to disorders in the quality of sleep such as abnormal sleep, or the normal rhythm of sleep and wakefulness alternately occur, the difficulty to start and maintain sleep, and excessive somnolence disease. Among these disorders, insomnia and obstructive sleep apnea syndrome (OSAS) are the most common. Insomnia is often characterized by difficulty falling or staying asleep when there is adequate opportunity for sleep and is associated with various adverse complications (4).

Sleep disorders can be evaluated by numerous methods(**Table 1**). Polysomnography is the gold standard for assessing sleep, mainly used for sleep and dream studies, as well as for the diagnosis of depression and OSAS. Polysomnography is to obtain biological signals through bioelectricity in different parts or through different sensors. After preamplification, the output is different electrical signals, which are collected, sorted and analyzed by microcomputer, to achieve the purpose of clinical diagnosis. However, its use in large studies assessing sleep is limited by the requirement that patients must be observed in a sleep laboratory (5). These days, the most popular methods for clinical evaluation of sleep are subjective patient reports, including the Pittsburgh Sleep Quality Index (PSQI) and the Athens Insomnia Scale (AIS). The PSQI comprises 19 self-evaluation and 5 additional evaluation items. However, in the final score calculation, the 19th self-evaluation item, and the 5 other evaluation items are not included. Each of the 18 self-assessment items has a score of 0–3 points, with a total score of 0-21. A higher PSQI score indicates poorer sleep quality. It is commonly used to evaluate sleep quality in the past month (6). The AIS, developed using the 10th International Classification of Diseases and Related Health Problems, is a valuable tool for evaluating subjective insomnia. The eight elements on the scale can be scored from 0 to 3, where 0 means “No problem at all” and 3 signifies “Very serious problem.” The total score is 0 to 24, with a score of 6 or above associated with insomnia (7).Other tools include Actigraphy, Sleep monitoring, and the Patient-Reported Outcomes Measurement Information System(PROMIS). Actigraphy measures sleep by monitoring the nocturnal movements of a patient using a device worn by the patient (5). However, it requires purchasing monitors for patients to wear, and is therefore, less commonly used for sleep studies at large hospitals. Due to the significant subjective influence of patients on the above evaluation methods, it is necessary for sleep doctors to conduct professional evaluations and objective measurements in clinical practice to obtain more reliable diagnostic results.

**Table 1:** Common screening methods for sleep disorders.

Methods	Advantages	insufficient	Author(Reference)
Polysomnography	Non-invasive Comprehensive Accuracy Objectivity	High professional requirements Limitations on the subjects being tested Time consuming Expensive	(5)
PSQI	Ease of use Comprehensive High reliability Efficient Wide application	Highly subjective Time limitations Content limitations Cultural Sensitivity	(6)
AIS	Simple Multidimensionality Efficient Wide application High reliability	Highly subjective Lack of specific etiological information Limited adaptability to specific populations	(7)
Actigraphy	Non-invasive Convenience Cost-effective Ease of use Objectivity	Lack of accuracy Limited adaptability to specific populations	(5)
Sleep monitoring	Objectivity Timely Personalized advice	Inconvenient use of equipment High professional requirements Expensive Privacy leakage	(8)
PROMIS	Standardization Consistency Comprehensive Patient centralization Flexibility	Implementation is difficult Affected by patient compliance Influenced by cultural differences	(9)

PSQI, Pittsburgh Sleep Quality Index; AIS, Athens Insomnia Scale; PROMIS, Patient-Reported Outcomes Measurement Information System.

## 3 Prevalence

Sleep disorders, arising during different phases of cancer diagnosis and treatment, rank among the most excruciating symptoms endured by cancer survivors. According to a Swedish study, 20–30% of adults frequently suffer from sleep disorders, and cancer survivors are 2–3 times more likely to experience sleep disorders (10).

The incidence of sleep disorders in various literature and reports differs widely. Davidson found in a large sample that about one-third of patients with cancer experience excessive sleepiness and insomnia, and nearly one-fifth sleep more than usual (11), consistent with 30-50% sleep disorder prevalence among cancer survivors reported in study. In another survey, 52% of cancer survivors reported to have trouble sleeping, and 58% said that their sleep issues were either due to or made worse by their cancer (12). A systematic review that included 89 studies found that the prevalence of sleep disorders in cancer patients was as high as 95% (13). This can be attributed to many factors, including limited sample numbers and disparate evaluation techniques, as well as variations in tumor forms, disease stages, and anti-cancer treatment approaches, all of which substantially affect the incidence rate. Insomnia symptoms were more prevalent in patients with malignancies of the gastrointestinal tract, gynecologic, lung, and breast, according to Davidson (11); this finding was supported by subsequent studies (10).As the cancer progresses, patients with sleep disorders may experience worsening of their condition, which may be worsened by the side effects from treatment. Sleep disorders are reported by 35% of patients with early-stage cancer and 45% of patients with advanced cancer. According to Savard, more than 50% of women who underwent radiation therapy complained of sleeplessness, and over 20% of these women satisfied the requirements for clinically severe sleeplessness (14). The study also reported that cancer survivors were still affected by sleep disorders long after treatment; one study indicated that sleep disorders affected 51% of cancer survivors 5 years after treatment (15).

It is worth noting that compared to the extensive literature on cancer, there are relatively few studies examining sleep disorders in patients with cancer. In addition, most studies used convenient small samples and relied on cross-sectional designs. Few studies have applied diagnostic criteria for insomnia to estimate prevalence in a randomized sample of patients with cancer, and most rely on one or two self-report scales. This has led to a high degree of heterogeneity in the prevalence of sleep disorders between reports.

## 4 Risk factors for sleep disorders in patients with cancer

Patients with various types of cancer frequently experience sleep disorders. Cancer-related sleep disorders, including insomnia, hot flashes, sleepiness, and nightmares, are frequently brought on by the psychological fallout from receiving a cancer diagnosis, and the direct effects and adverse effects of cancer treatment. The exact cause of sleep disorders resulting from cancer remains mostly unknown due to the variability in cancer types, available treatments, patient demographics, and lifestyle choices.

### 4.1 Disruption of biological rhythms

The circadian clock regulates sleep, and the central clock of the brain serves as a pacemaker for other molecular clocks in the peripheral organs, synchronizing sleep with external cues like light and food availability. The sleep wake cycle is determined by the inherent biological clock or circadian rhythm. The sleep phase occurs in a repetitive pattern or cycle of non-rapid eye movement sleep (NREM) and rapid eye movement sleep (REM), with each cycle lasting approximately 90 minutes (16). The physical illness, pain, treatment, and psychological effects of cancer may disrupt the sleep patterns of cancer patients (17). Disruption of individual sleep patterns can disrupt circadian rhythms, impair sleep cycles, and ultimately lead to a variety of human diseases, including sleep disorders, mental and neurodegeneration disorders, cardiovascular disease, and cancer (18–20).The relationship between circadian rhythm, sleep patterns, and exhaustion in patients with cancer, however, is still unclear.

### 4.2 Diagnosis and treatment of cancer

The diagnosis and treatment of cancer seem to be associated with high levels of insomnia symptoms. One such example is the population with ovarian cancer, where the most common risk factor for sleep disorders is severe pain upon diagnosis. Tumor physiology itself may also play an important role here, such as the tumor or tumor microenvironment’s secretion of cytokines affecting metabolism and energy balance during sleep. According to a large longitudinal population survey, 31% of patients experience symptoms of insomnia in the perioperative period, and nearly 30% of patients match the diagnostic criteria for insomnia syndrome. In that study, 2 months later, the insomnia symptoms were relieved, and the remission rate was 32% (21).In another study, cancer survivors were found to be more likely to report sleep disorders after 2 years (22). A recent longitudinal study of American women’s health pointed out that among middle-aged women diagnosed with breast cancer, there was no difference in sleep problems before and after diagnosis (23).

Sleep problems often arise after surgical intervention. Poor postoperative sleep quality was present in over 70% of patients with cancer compared to baseline, and this condition tended to continue post-therapy (24). Body image disorder after treatment may be a significant predictor of poor sleep quality in patients receiving radical surgery. Beesley et al. noted that sleep duration but not sleep quality of patients with cancer improved 6 months after surgery (25). According to that study, more optimistic people typically felt less depressed and had better sleep. While neoadjuvant and adjuvant chemotherapy are frequently used along with surgery, which is the primary treatment for many malignancies, these treatments can also interfere with sleep. In women with cancer receiving palliative chemotherapy, one of the most common adverse effects was sleep disturbance, accounting for 24% of the outcome measures recorded, according to King et al. (26). Palesh found that 79.6% of patients in cycle 1 had insomnia symptoms during the chemotherapy phase in patients with different types of cancer, with a slight decrease in symptoms of insomnia by cycle 2 (10). Some patients experience good sleep in the first cycle of chemotherapy and exhibit insomnia symptoms in the second cycle. Sleep disturbances may arise in as many as 65% of patients undergoing adjuvant chemotherapy and radiation therapy (27). Research has found that up to 60% of prostate cancer patients receiving androgen deprivation therapy combined with radiotherapy experience sleep disorders (28). Additionally, the percentage of patients with cancer who fulfill the diagnostic criteria for insomnia is three times greater than that of the general population (10). Most cancer survivors getting combination therapy had sleep disturbances. Receiving therapy can be a difficult procedure, and medication-induced side effects are the most common cause of treatment-related sleep disorders. Patients treated for cancer often experience stress, pain, anxiety, depression, and higher levels of physical fatigue. One of most common causes of sleep delays or interruptions is pain because it can persistently disrupt sleep throughout the night (29), particularly in patients suffering from chemotherapy-induced peripheral neurotoxicity. In turn, sleep disruption further leads to hyperalgesia (30), forming a vicious cycle of mutual influence between the two. Fear of a cancer diagnosis, uncertainty about the therapy outcome, and fear of recurrence and death are the main causes of anxiety and depression. These may affect sleep through intrusive or focused thinking. Depression levels and cancer duration are important predictors of sleep quality. Cancer-related physical fatigue is often persistent during treatment and manifests as reduced motivation to perform daily chores such as laundry, work, and cooking (31). This physical fatigue is not diminished by sleep or rest. Sleep disorders in patients with cancer may be more severe in patients with cancer-related fatigue. Treatment-induced nausea, vomiting, urodynamic changes, hot flashes and respiratory problems often lead to sleep disruption (32). Women are more likely to experience nocturia and hot flashes, potentially interfering with their ability to fall back asleep. A deterioration in sleep quality during diagnosis, treatment, and after treatment may be due to shorter sleep duration overall and more awakenings.

### 4.3 Others

Breast cancer has been linked to the highest incidence of sleeplessness when compared to other cancers, such as those of the prostate, gynecological, head and neck, urinary, or gastrointestinal. It characterizes low sleep efficiency, frequent wakefulness at night, long wakefulness after sleep, and daytime sleepiness. Besides the physical discomfort, pain, and hot flashes caused by breast cancer itself, the patient is highly uncertain about the treatment outcome. Concerns about body shape and hormonal changes caused by hormonal therapy can lead to sleep disorders in patients. Koopman et al. discovered that 63% of women with metastatic breast cancer had sleep difficulties (33), indicating that in advanced cancer, patients are more prone to experience these problems. Further risk factors for sleep disorders are women, young age, less education, less physical activity, vasoconstriction (particularly night sweats), reproduction, less social support, higher levels of social stress, life events, and external factors like light, noise, or interference from hospital staff during hospital stays or sleep-inducing nursing interventions (10).

## 5 The impact of sleep disorders in patients with cancer

There exists a complex, two-way, chicken-or-egg link between sleep disorders and cancer. Cancer seems to cause sleep disorders, which, in turn, contribute to the development and progression of cancer (34).

### 5.1 Sleep disorders and cancer risk

Recent research reports suggest that individuals with sleep difficulties or diagnosed with sleep disorders at any point in their lives are more likely to be diagnosed with cancer (35), and sleep disorders can be explained as early symptoms and indications of tumor diseases, predicting short-term health outcomes (36). Studies have found that sleep disturbances may increase the risk of gastric, colorectal, and breast cancers, and the risk of cancer increases with the duration and severity of sleep disorders. In the prospective AGES-Reykjavik cohort study, it was found that men with sleep disturbances had a considerably higher risk of prostate cancer than those without sleep disruption (37). Research has found that both insufficient sleep and prolonged sleep can increase the risk of cancer, with people who sleep longer having a 22% higher risk of cancer compared to those who sleep for a normal number of hours (7 to 8 hours). Similar findings were obtained in patients with colorectal cancer(CRC). The study found that abnormal sleep patterns, including nocturnal timing, may also be linked to skin, colorectal, and ovarian cancer. Overall, nocturnal patterns, symptoms of insomnia, and excessive and insufficient sleep were highly predictive of an elevated risk of cancer. Lack of sleep can lower the body’s immune function and increase the risk of cancer. Long-term sleep has been associated with an elevated risk of cancer, with prior research indicating that it can negatively impact metabolite levels, neurocognitive function, and cardiovascular health (38). However, additional research is required to identify the potential pathophysiological pathways involved in extended sleep duration and increased cancer risk. Patients with a nocturnal pattern are at a higher risk for cancer, possibly with an additional burden of nocturnal pattern-induced circadian delay worsening the loss of circadian autonomic regulation of tumor cells, causing dyssynchrony in the circadian control of cellular metabolism (39). The study discovered that the sleep disorder and cancer risk-association may be mediated by the combined effects of age, occupation, and sleep disorders.

Another prevalent sleep issue among patients with cancer is OSAS. OSAS is a very common sleep breathing disorder, but it is severely underestimated in clinical practice. The pathophysiological processes related to OSAS, such as fragmented sleep and intermittent hypoxia, may affect normal neuroendocrine regulation and impair the body’s immune function (40). The associations of cancer at different anatomic sites and/or different pathologies with OSAS may differ. Studies have indicated an association between OSAS and elevated cancer incidence and mortality (41, 42), especially head and neck cancer (43), and its severity is an independent risk factor for cancer. In addition, peripheral inflammation may also be involved in the carcinogenic mechanism of OSAS. However, some studies have not discovered a strong correlation between OSAS and cancer (44, 45).

### 5.2 Sleep disorders and cancer cell proliferation and migration

Sleep disturbances can encourage cancer cells to proliferate and migrate. It is likely that sleep disorders-induced circadian rhythm disturbances, decreased melatonin secretion, and inflammatory responses result in unregulated cell proliferation. Circadian disruptions can influence the expression of genes linked to tumor development and metastasis, DNA repair, cell cycle regulation, and apoptosis (46). Circadian dysregulation may also attenuate the efficacy of anti-cancer therapy (47). The World Health Organization currently lists circadian rhythm abnormalities as potential cancer-causing agents. Melatonin is the first nocturnal anti-cancer signal discovered in humans (48), which is affected by circadian disturbances and ambient light. Chen et al. showed melatonin to have a positive anti-cancer impact by preventing the formation of lung cancer in a Lewis animal model (49). One cross-sectional investigation confirmed the findings and discovered considerably lower melatonin levels in malignant non-small-cell lung carcinoma than in healthy volunteers. According to animal studies, the number of cytotoxic cells and the capacity of the immune system to react to tumor growth are significantly impacted by lack of sleep. Lack of sleep also promotes proinflammatory cytokine and induces an inflammatory microenvironment (50), and persistent inflammation leads to genetic instability, causing cell mutations. These may promote cancer onset and progression. Sleep deprivation can also raise peripheral blood gamma-aminobutyric acid (GABA) levels and promote the expression of miR-223-3p. Extracellular miR-223-3p promotes the polarization of M2-like macrophages by activating the macrophage mitogen-activated protein kinase pathway, increasing interleukin-17 (IL-17) secretion, and promoting tumor proliferation and migration (51).

Several studies have reported a 3.4-to 4.8-fold increase in mortality in patients with cancer having OSAS (41, 42). It is most likely because of the reconfiguration of the cytoskeleton and connexin brought on by intermittent hypoxia that endothelial barrier failure increases tumor cell escape and metastasis. Moreover, hypoxia causes angiogenesis and vascular development by enhancing the expression of angiogenic growth factors released by tumor cells and endothelial cells (52). In lung cancer patients, OSAS promotes the activation and enrichment of cancer associated fibroblasts (CAFs), hypoxia activates TGF β signaling in lung cancer cells and fibroblasts, and promotes epithelial mesenchymal transition (53). Recent studies have found that intermittent hypoxic environments stimulate cell proliferation and migration, which may be associated with increased miRNA expression and HIF-1 (54).

### 5.3 Sleep disorders, anxiety, and depression symptoms

Epidemiological statistics indicate a correlation between anxiety and depression and a higher likelihood of sleep problems. Conversely, those who already have sleep disorders typically exhibit greater symptoms of anxiety and despair; There may be a vicious circle between the two. A substantial amount of evidence indicate that sleep disorders are independent risk factors for depression in healthy individuals and older individuals (55), as well as powerful predictors of depression and relapse in those with a history of depression (55, 56). On the other hand, survivors with better sleep had higher levels of happiness and fewer episodes of anxiety or sadness. A correlation between sleep disturbances and an inflammatory reaction to depression has been shown (56).

### 5.4 Sleep disorders and cognition, memory

A direct link between sleep disorders and memory problems in humans and animals has been often shown (57). Sleep disorders in humans can impair motor programming, implicit memory, and working memory. There exists a strong correlation between sleep disorders and cognitive decline, as well as an elevated risk of postoperative delirium. This may be related to insufficient sleep, leading to neuronal damage and accumulation of waste products in the brain (58).

### 5.5 Sleep disorders and quality of life, survival rate

Research has found that sleep problems in cancer patients are associated with lower clinical prognosis and quality of life (59). Adequate sleep can increase the pain tolerance of cancer patients, while poor sleep may lead to increased postoperative pain, delayed healing, prolonged postoperative recovery and hospital stay, suboptimal treatment outcomes and interruptions, and decreased ability to perform daily tasks. These lead to a decline in quality of life (10, 15). Epidemiological studies report that untreated insomnia leads to associated increase in absenteeism, decreased workplace productivity, and work-related as well as motor vehicle accidents. Sleep disorders remain an independent predictor of cancer mortality, even after adjusting for variables including age, cortisol levels, estrogen receptor expression, and comorbid depression (3). According to Ren Shave, for every 10% increase in sleep efficiency, there is a 32% decrease in mortality. Studies found that both short (≤6 hours/day) and long (≥8 hours/day) sleep durations increase the risk of mortality compared with sleep of 7 hours/day (60, 61). This has also been confirmed in several recent large-scale studies (62, 63). New research suggests that sleep regularity can predict mortality more strongly than sleep duration. The higher the sleep regularity, the lower the risk of all-cause mortality by 20-48% and cancer mortality by 16-39% (64). This may be related to irregular sleep as a more direct measure of circadian rhythm disorder.

### 5.6 Sleep disorders and others

Excessive daytime sleepiness and sleep disturbance impact fatigue and its perception. Sleep disorders are thought to be a cause of cancer-related fatigue and often accompany cancer-related fatigue (65). Furthermore, circadian dysregulation increases the risk of obesity and associated metabolic disorders, such as diabetes, hypertension, inflammation, and cardiovascular disease (66). These increase the risk of cancer and adversely impact societal health and the economy.

## 6 Treatment

Patients with cancer frequently experience sleep disorders; therefore, prevention or treatment of sleep disorders, especially in the early stages of personalized treatment, can help reduce cancer-related pain, fatigue, and mental health issues like anxiety and depression. It is also possible to prevent the development, preservation, and/or deterioration of cancer, which can improve quality of life and survival rate of patients with cancer.

### 6.1 Medication

Pharmacological methods are among the most popular choices for treating sleep disturbances in cancer survivors, particularly in their acute stage. This is like the general population with sleep disorders. Common pharmacologic therapies include symptomatic medications (such as pain, diarrhea, and cough relief) and the use of benzodiazepine-based and non-benzodiazepine hypnotics, as proposed by Pillai et al. (67). After benzodiazepine receptor agonist treatment, 47.7% of the participants were actively treated for remission. However, such drugs are often restricted to short-term use because of their residual effects, tolerability, dependence, withdrawal, and potential risks of abuse, as well as changes in sleep architecture. Common medications include antidepressants, antihistamines, zolpidem, melatonin (68), and dexmedetomidine. Antihistamines have been found to lower the quality of sleep, but they also cause narcolepsy. Zolpidem can reduce postoperative pain and fatigue, improve patients’ quality of life, and reduce the consumption of anesthetic drugs. Dexmedetomidine has the unique advantage of simulating a natural sleep state, making it easier for patients to wake up, minimizing respiratory impact, improving their cognitive function, and reducing the incidence of postoperative delirium (68). Mechanistically speaking, on the one hand, dexmedetomidine can weaken neuronal apoptosis and have a protective effect on neuronal damage. However, it can also lower norepinephrine (NE) release, greatly decreasing the interstitial space’s extracellular volume and speeding up the brain’s waste-removal process (69). Dexmedetomidine is commonly used in Patient Controlled Sleep (PCSL). PCSL, a technology that allows patients with chronic intractable insomnia to intermittently trigger measurable doses of dexmedetomidine through a patient-controlled device to produce and maintain natural sleep (70). However, its application is challenged considering its long treatment time (possibly up to 6 months), inconvenient patient mobility, increased risk of infection, uncertainty in the efficacy of dexmedetomidine alone, and potential side effects of long-term use. Ozone is a strong oxidizing agent, which was first used to prevent wound infections in soldiers during World War I (71). Studies discovered that three months of low-dose ozone therapy decreased anxiety, sadness, and sleep quality metrics and significantly raised serum levels of BDNF and GABA in individuals with insomnia (72).A recent study have found that propofol may be a potential treatment for chronic sleep disorders, which may be associated with REM sleep recovery, enhanced mPFC functional connectivity, and improved brain metabolism (73). Over time, clinical doctors have realized the necessity of multimodal sleep by adopting personalized treatment methods that integrate different drugs delivered by patient-controlled fusion pumps, improve sleep/wake cycles, treat complications caused by chronic insomnia, and provide vital signs and sleep monitoring. Among the medications utilized are lidocaine, scopolamine, sodium 4-hydroxybutyrate, propofol, ketamine, and dexmedetomidine. Alternatively, transcranial magnetic stimulation and stellate ganglion block are used (74). Multimodal sleep improves physical and emotional well-being of patients with cancer, and thus has become a commonly used treatment for refractory insomnia in large hospitals.

Although drug therapy has been widely attempted and considered beneficial for sleep, guidelines are still recommended for its short-term use (2–4 weeks), combined with long-term lifestyle changes and immediate management. Drugs cannot reconstruct normal sleep structures while improving sleep quality. Generally, it is helpful for any form of insomnia in the short term. If taken for too long, sleep regulation will be externalized, and once stopped, it is likely to rebound. In addition, long-term use of hypnotic drugs can lead to cognitive deterioration, increased fall and fracture rates (75).

### 6.2 Non-drug therapy

Patients with cancer frequently experience chronic sleep disturbance, and nonpharmacologic therapy may be helpful since it reduces the possible negative effects of long-term drug use. Common methods include CBT, exercise, massage, and relaxation therapy and improve the patient’s sleep environment.

#### 6.2.1 The role of cognitive behavioral therapy

Studies have shown that CBT provides lasting improvement in insomnia, and the American College of Physicians recommends CBT-I as a first-line treatment (76, 77). Use of CBT is effective in improving functional health, psychological well-being, and sleep-related quality of life in people reporting insomnia symptoms (78). Notify patients in advance of common side effects and coping measures of treatment to improve their self-management ability. Studies show that gynecological or breast surgery, CBT can be useful in enhancing sleep efficiency, overall awakening time, and sleep onset time in patients. Since CBT-I usually lasts long after treatment ends, it may positive effects may last longer than medication. However, Morin et al. showed that using CBT-I alone resulted in only 40% of patients receiving positive relief from the treatment (79).

#### 6.2.2 Exercise therapy

Exercise, which includes yoga, walking, and other non-pharmacological treatments, is thought to help patients with cancer sleep better. It has also been demonstrated to lessen fatigue in patients with cancer receiving chemotherapy (80). Further, exercise is particularly effective when combined with CBT. Exercise also plays a significant role in preventing and treating cancer by promoting immune cell infiltration, regulating the tumor microenvironment, promoting skeletal muscle factor secretion and increasing circulating levels of β - endorphins.

#### 6.2.3 Massage and relaxation therapy

Massage and relaxation therapy is also one of the non-drug treatment methods. A recent study found that acupuncture and massage can help relieve pain in patients with advanced cancer, improve fatigue, insomnia and quality of life (81).Additionally, it has been demonstrated that therapeutic massage greatly enhances the quality of sleep in fibromyalgia patients (82). Patients with cancer may have easier access to massage and relaxation therapy than they have to CBT, exercise therapy, or even people with advanced cancer. Therefore, more research is needed to evaluate how these approaches can be better integrated into pain management.

#### 6.2.4 Photobiomodulation therapy

Recent evidence suggests that PBM, and specifically intravascular laser blood irradiation (ILIB), can significantly improve insomnia (83). It is possible that ILIB can reduce pain and discomfort and lower creatine levels (83), regulate immunity (84), promote tissue recovery and reduce inflammation (85). The study found that the use of office-based laser near-infrared transcranial photobiomodulation (tPBM) of power density 250 mW/cm^2^ in elderly participants aged 50–79 years, could significantly improve the sleep quality of participants after only 5 days of treatment (86). In addition, tPBM has been shown to improve the cognitive impairment associated with sleep deprivation (87). A recent study reported that a wearable, self-administered continuous tPBM treatment was effective in improving sleep quality (88). PBM is expected to be a new, safe and effective alternative therapy in the future.

#### 6.2.5 Improve the sleeping environment

The sleep environment is also crucial to improving sleep quality, especially during hospitalization. The two most common things that disrupt sleep are light and noise. A well-established sleeping environment is dark, quiet, and comfortable, which can effectively promote sleep quality. The common interventions include earplugs and eye masks. These can filter out light and noise, lengthen and deepen sleep, lower pain thresholds, and lessen the frequency of nighttime awakenings (68). It has also been demonstrated that minimizing overnight disruptions by lowering midnight care activities and carrying out centralized care activities enhances the quality and length of sleep (10, 68). The National Cancer Institute recommends several interventions specifically related to hospital care, including regulating fluid intake, encouraging patients to urinate before bed to avoid waking up at night to urinate, relaxing before bed, and avoiding caffeine or other stimulants. Further, patients were encouraged to get out of bed and move as much as possible during the day. Patients with cancer can effectively improve their quality of sleep and reduce fatigue due to the disease by implementing these treatments at low or no cost. Bright white light therapy (BWLT) uses high-intensity (10000 lumens) household fluorescent lamps to stimulate the suprachiasmatic nucleus of the hypothalamus, which regulates circadian rhythms, in order to treat emotional depression and sleep disorders. In patients with ovarian and endometrial cancer disorders, BWLT has been demonstrated to improve sleep disorders and quality of life (89), especially in older patients, which can lead to longer sleep (90). BWLT is often used in patients who still have sleep disorders after discharge from the hospital.

#### 6.2.6 0ther approaches

In the past few years, music therapy has received great attention in patients with sleep disorders and depression due to its non-invasive nature and minimal side effects. It improves sleep by regulating cortisol, releasing dopamine, and soothing autonomic nervous system (91). Furthermore, long-term medical care for patients with cancer includes a web-based telehealth program (92). A recent study found that using sleep wearable devices to provide sensor based feedback and guidance on sleep parameters can reduce the severity of insomnia and sleep disorders (93).

Good sleep helps patients with cancer have a better prognosis and quality of life, so addressing their sleep issues at the time of diagnosis, throughout therapy, and after treatment is crucial. The sleep scale should be used regularly by clinicians to evaluate patients’ sleep while screening for possible sleep disorders, such as prolonged sleep latency, frequent wakefulness and decreased sleep efficiency, fatigue, and drowsiness during the day, loud snoring, inappropriate behavior at night, and/or nightmares. It is worth noting that anxiety and depression in patients with cancer are important factors contributing to sleep disorders, and managing anxiety and depression seems to be necessary and should be the target of intervention. At present, the common treatments for anxiety and depression include antidepressant medication, psychological intervention, music therapy, recall therapy, CBT, and electronic health intervention. In addition, breathing problems during sleep should also be taken into account. For example, patients with lung cancer may experience inadequate ventilation during sleep (94), and it is very dangerous to prescribe drugs that reduces respiratory motility. Therefore, in clinical practice, sleep physicians and surgeons should jointly evaluate to determine the best way to solve the problem.

## 7 Underlying mechanisms of cancer and sleep disorders

### 7.1 Circadian rhythm

**Figure 1:**
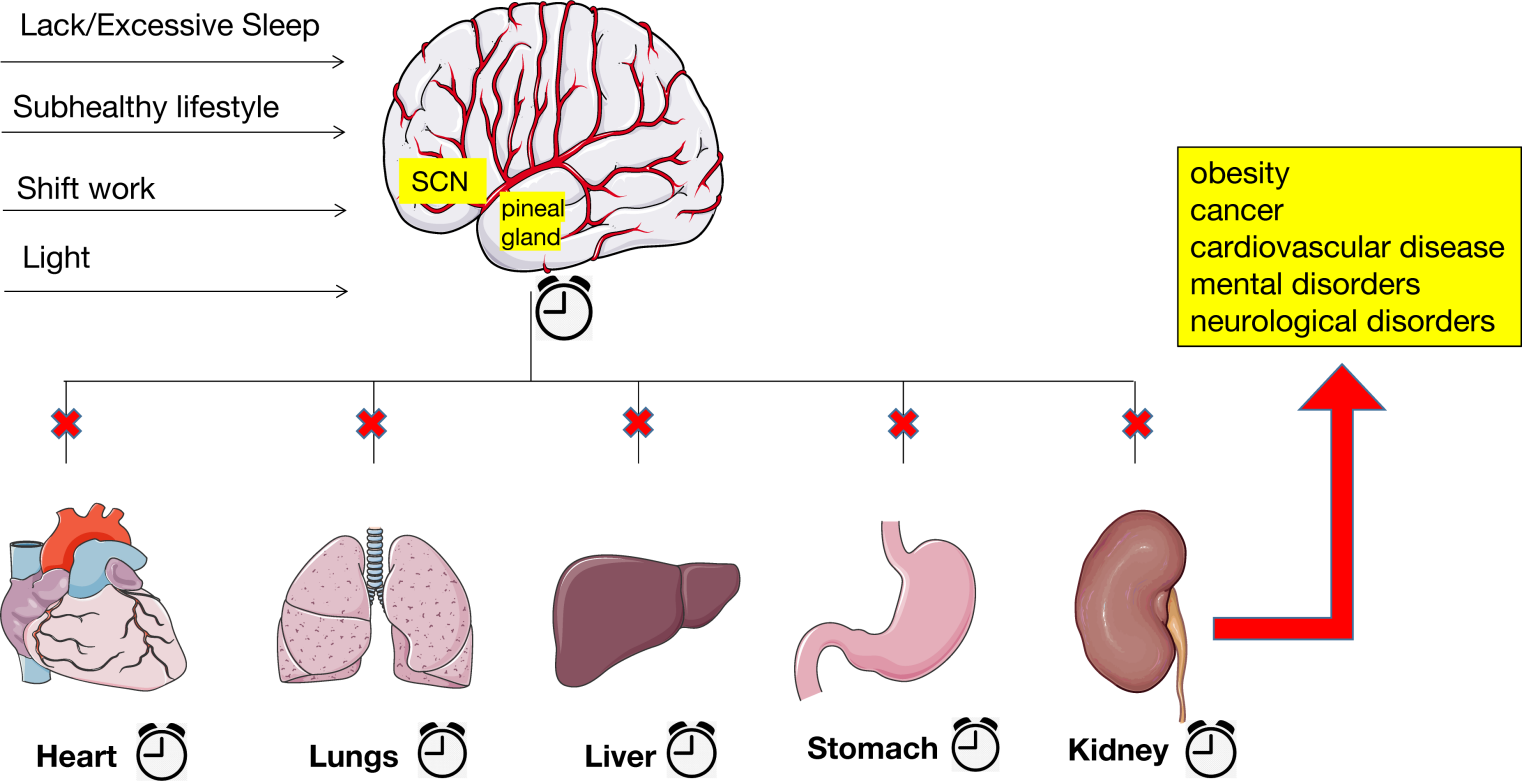
Alterations in the circadian rhythm lead to many kinds of diseases. Chronic sleep deprivation or excessive sleep, subhealthy lifestyle, shift work, and exposure to artificial and blue light at night have blurred the lines between day and night and raised the likelihood of disrupted circadian rhythms. The disruption of central and peripheral clocks, which causes extensive dysregulation of clock biological processes, ultimately leading to several diseases, such as obesity, cancer, cardiovascular disease, and mental and neurological disorders.

Endogenous cycles in physiology, hormones, and behavioral processes known as circadian rhythms regulate physiological and behavioral activities of each organ to maintain systemic homeostasis. Several key biological processes are regulated by circadian rhythms, including the sleep-wake cycle, food-fast cycle, and activity-rest cycle. Among them, the sleep-wake cycle is most likely the most well-known biological process that adheres to a 24-hour circadian rhythm (95). It provides the foundation for preserving the body’s optimal functioning and is governed by the interaction between endogenous circadian rhythms and steady-state sleep drivers (96). The sleep-wake cycle is strongly bidirectional to the circadian system, with changes in one system affecting the other. Disruption of the sleep-wake cycle, such as chronic sleep deprivation, can disrupt the circadian rhythm. While acute circadian rhythm disruptions can be uncomfortable for a short while, chronic circadian rhythm disruptions can impair the body’s ability to function. This can cause several diseases, such as obesity, cancer, cardiovascular disease, and mental and neurological disorders (**Figure 1**) (97). Circadian clock genes, including clock, PER, and BMAL1, are impacted by mutations or deletions that impact blood pressure, endothelial function, and glucose homeostasis. Sleep-deficiency-dysregulated CLOCK hypertransactivates ACSL1 to stimulate fatty acid oxidation, mitochondrial respiration, and ATP production, thereby promoting cancer progression (98).

On the other hand, the Circadian clock is essential for cell cycle regulation, and changes in clock function can lead to abnormal proliferation, growth, and DNA damage in cancer cells (99). A large number of epidemiological and animal research have demonstrated that a persistent disruption of circadian rhythm promotes the establishment of cancer characteristics and raises the risk of both breast cancer and prostate cancer (100). Modern culture and lifestyle trends have blurred the lines between day and night and raised the likelihood of disrupted circadian rhythms. These trends include exposure to artificial and blue light at night, longer working hours, shift employment, and an all-weather lifestyle (101). Studies have indicated that women who work night shifts are almost 50% more likely to develop cancer compared to women who do not work night shifts. The International Agency for Research on Cancer reclassified shift work involving circadian rhythm disorders as “possible human carcinogens” (Group 2A) in 2019, based on the research of human and animal cancer mechanisms (102). In 2021, the National Toxicology Program concluded that “continuous night shift work” and “certain lighting conditions” have post-cancer risks (102). Furthermore, the formation of tumors directly weakens the brain’s control over rhythms (51). The disruption of central and peripheral clocks, which causes extensive dysregulation of clock biological processes and melatonin inhibition, is thought to raise cancer risk due to the controlled disruption of circadian rhythms (97). Given these observations, using biological clock components as new targets for chronic diseases such as cancer have attracted widespread attention (103). In theory, there are two pharmacological methods; one is to regulate the core genes of circadian rhythm directly (104), but this is currently still very challenging. Another approach consists of medication formulations targeting proteins—such as REV-ERBα/β, RORα/β/γ, CRY1/2, casein kinase family, and FBXL3—that phosphorylate or degrade clock components. The molecular understanding of circadian rhythms has opened up new frontiers in cancer treatment, and drug regulation of the biological clock and biological clock therapy for cancer may become a new treatment option for better cancer management.

### 7.2 Neuroendocrine alterations

The hypothalamus is an essential component of the brain consisting of multiple nuclei that create and preserve systemic physiological equilibrium. It is critical for regulating sleep/wake behavior, body temperature, sexual behavior, appetite and completion stages of eating, system energy balance, and circadian rhythms (105). Hypocretin/Orexin (HO) neurons are found in the lateral region of the hypothalamus that are highly significant in regulating wakefulness and metabolism. This is because they can receive and integrate signals from the periphery about energy balance and immunological status (106). HO neurons can innervate multiple autonomous output nuclei in the brainstem and can alter the overall energy balance by signaling through the sympathetic nervous system (SNS).Additionally, the Hypothalamus-pituitary axis (HPA) axis is involved in the induction of glucocorticoid production by HO neurons. Glucocorticoids have pleiotropic effects on the immune system and may have peripheral physiological effects on hyperactive states (e.g., anxiety, dread, panic, insomnia). In the context of cancer, many of the physiological signals sensed by hypothalamic neurons are frequently altered; these include inflammatory cytokines/chemokines, including interleukin (IL-6), glucose, and endocrine hormones, such as leptin and ghrelin (107). Increased ghrelin sensitivity, decreased leptin concentrations, elevated levels of IL-6 and blood glucose, and hyperactivity of HO neurons (increased cFos immunoreactivity) were observed in cancer mouse models during the development of sleep debris in the late phase of tumor growth (34).

On the other hand, dual-receptor antagonism promoted deep restorative sleep in tumor-bearing mice, which reduced the HO signal. The aberrant activity of these neuronal populations could be attributed to sleep disruption due to altered immunological, metabolic, or endocrine functioning caused by cancer. McAlpine and colleagues have shown that long, scattered sleep dramatically reduces the number of HO neurons in the lateral hypothalamus, a phenotype associated with arteriosclerosis development. Sleep and memory disorders in patients with cancer may be exacerbated by the dysfunction of melanin-concentrating hormone (MCH)-promoting neurons, which are susceptible to peripheral inputs that become unregulated (106). There is evidence that HO and MCH neurons have inhibitory feedback *in vitro*. The study found that gabaergic neurons in the preoptic ventrolateral area (Vlpo), Ventral tegmental area dopaminergic neurons, 5-hydroxytryptamine (5HT) neurons in the raphe nucleus, Locus coeruleus (LC) noradrenaline neurons and cholinergic signals in the brainstem are all involved in the development of cancer-related sleep disorders (106). Sleep in patients with cancer can also be affected by astrocytes (108), the astrocyte mediates sensory gain through distinct patterns of astrocytic Ca^2+^ transients, thereby contributing to the overall balance of sleep, wakefulness, and arousal states (109).

Functional alterations of the HPA axis are a common phenomenon in patients with cancer, characterized mainly by elevated corticotropin-releasing hormone (CRH) secretion adrenocorticotropic hormone(ACTH), which leads to abnormally high levels of glucocorticoid and catecholamine. Elevated cortisol can suppress the body’s immune function. According to a large number of studies, dysfunction of the HPA axis in cancer patients, such as pituitary tumors, neurosurgery and radiation therapy of the tumor, can cause symptoms such as depression, anxiety, and sleep disorders (110). Conversely, fatigue, depression, and sleep problems can also trigger HPA axis dysregulation, increase cortisol secretion, and impair the body’s immune function, thereby promoting tumor growth and metastasis. According to Wilkinson et al., lack of sleep is linked to a temporary rise in the autonomic sympathetic adrenal system and HPA activity (111).

Tumors modify the activity of cells in both their immediate and distant environments to avoid detection by the immune system and meet the metabolic needs for growth, multiplication, and metastasis. These cells include T cells, fibroblasts, and macrophages in the liver and brain (46). Chemokines that attract leukocytes, such as C-C motif chemokine ligand 2 (CCL2), CCL4, CCL5, CCL7, CCL8, and CCL20 (112), can be released by cancer cells and their microenvironments, along with a variety of growth factors secreted by leukocytes. These factors can frequently reach the entire body through neural and/or humoral pathways to promote abnormal neural activity (113). This can result in fatigue, disruptions to sleep and circadian rhythms, energy balance issues, inflammation, reduced food intake, cachexia/anorexia, and, most commonly, sleep disruption (114). In turn, sleep disorders may disrupt these balances and affect the secretion of factors at specific times. Cancer-induced depression has also been found to be associated with growth hormone and insulin-like growth factor-1 secretion (115).

### 7.3 Immune system and inflammation

**Figure 2:**
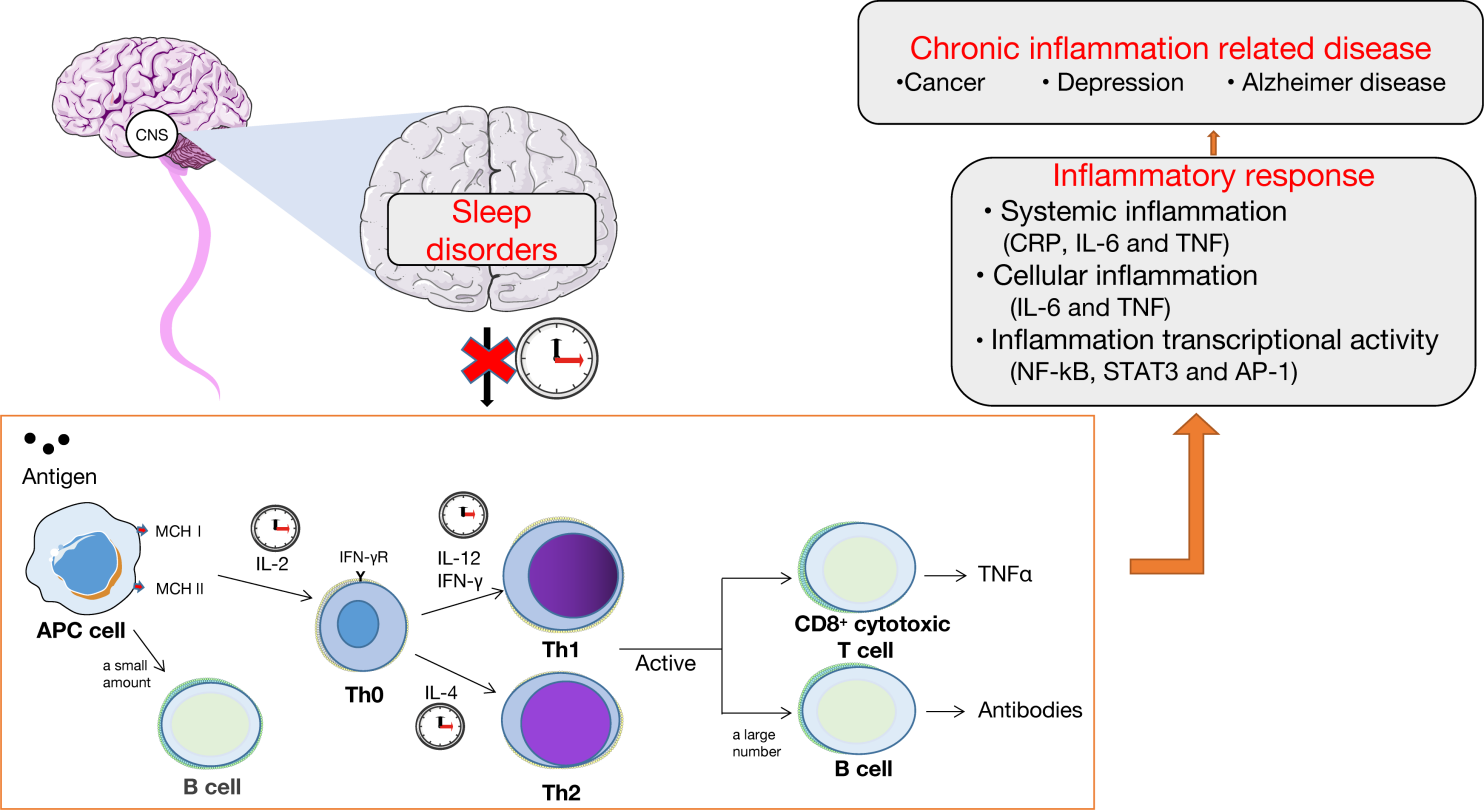
Sleep disorders, immune system, and inflammation. The normal sleep cycle maintains the normal function of the immune system by regulating the balance of the expression of cytokines, which is beneficial to the transfer of antigens. Chronic sleep disorders disrupt the circadian rhythm, affecting the normal function of the immune system, leading to inflammatory reactions in the body, including systemic inflammation, cellular inflammation, and inflammatory transcriptional activity, ultimately leading to chronic diseases such as cancer and depression.

Adequate sleep is crucial for maintaining strong immune function, which plays a key role in cancer prevention through mechanisms such as immune surveillance (116). Sleep disorders can lead to immune system disorders and abnormal activation of inflammatory responses (117, 118), thereby promoting tumor growth and metastasis. Sleep plays a crucial role in regulating the immune system, with increased production and release of cytokines such as IL-1 and tumor necrosis factor (TNF) during sleep. Sleep also promotes the proliferation of T cells and strengthens the interaction between dendritic cells and T cells. Sleep affects the balance between pro-inflammatory and anti-inflammatory responses. Under normal circumstances, the immune system maintains normal immunological function by balancing T-helper Factor 1 (Th1) and Th2, cell-derived cytokines (Th1 cytokines: IL-2, IFN-γ, and IL-12; Th2 cytokines: IL-4 and IL-10), preferring the antigen delivery pathway (119). During specific periods of nighttime sleep, these cytokines are secreted, such as Th1 activity, which is often enhanced during the first few hours of nighttime sleep, and Th2 activity, which often peaks late in sleep or before awakening (120). This pattern is broken by sleep disorders, with excessive production of cytokines causing immune disturbances, attenuated antigen processing, and antibody formation (121), ultimately causing chronic inflammation. The abnormal activation of the inflammatory response is associated with increased levels of inflammatory factors like TNFγ, IL-6, and c-reactive protein, as well as the activation of inflammatory signals like nuclear factor-kappa (NF-ĸB), signal transducer and activator of transcription 3, and activator protein-1. It is also characterized by abnormal systemic, cellular, and inflammatory transcriptional activity (122). The immune system plays a crucial role in cancer prevention and development through its ability to monitor and eliminate malignant cells. Inflammatory response can promote the development, progression, and recurrence of chronic diseases such as cancer and depression (**Figure 2**) (123).In turn, anti-cancer therapy of the cancer or the cellular and/or tissue level leads to increase the release of proinflammatory cytokines and dysregulation of the circadian system (124). Increased release of pro-inflammatory factors is associated with increased pain scores in patients with cancer, often associated with severe sleep disorders and depressive states. Likewise, impaired CS can lead to decreased ability to perform daily chores, insomnia, reduced appetite, depression, and fatigue in patients with cancer. Depression can also promote tumor invasion and metastasis by inducing NF-ĸB activation and the HPA axis hyperfunction to coordinate the production of inflammatory mediators and systemic inflammation.

### 7.4 DNA damage and repair

**Figure 3:**
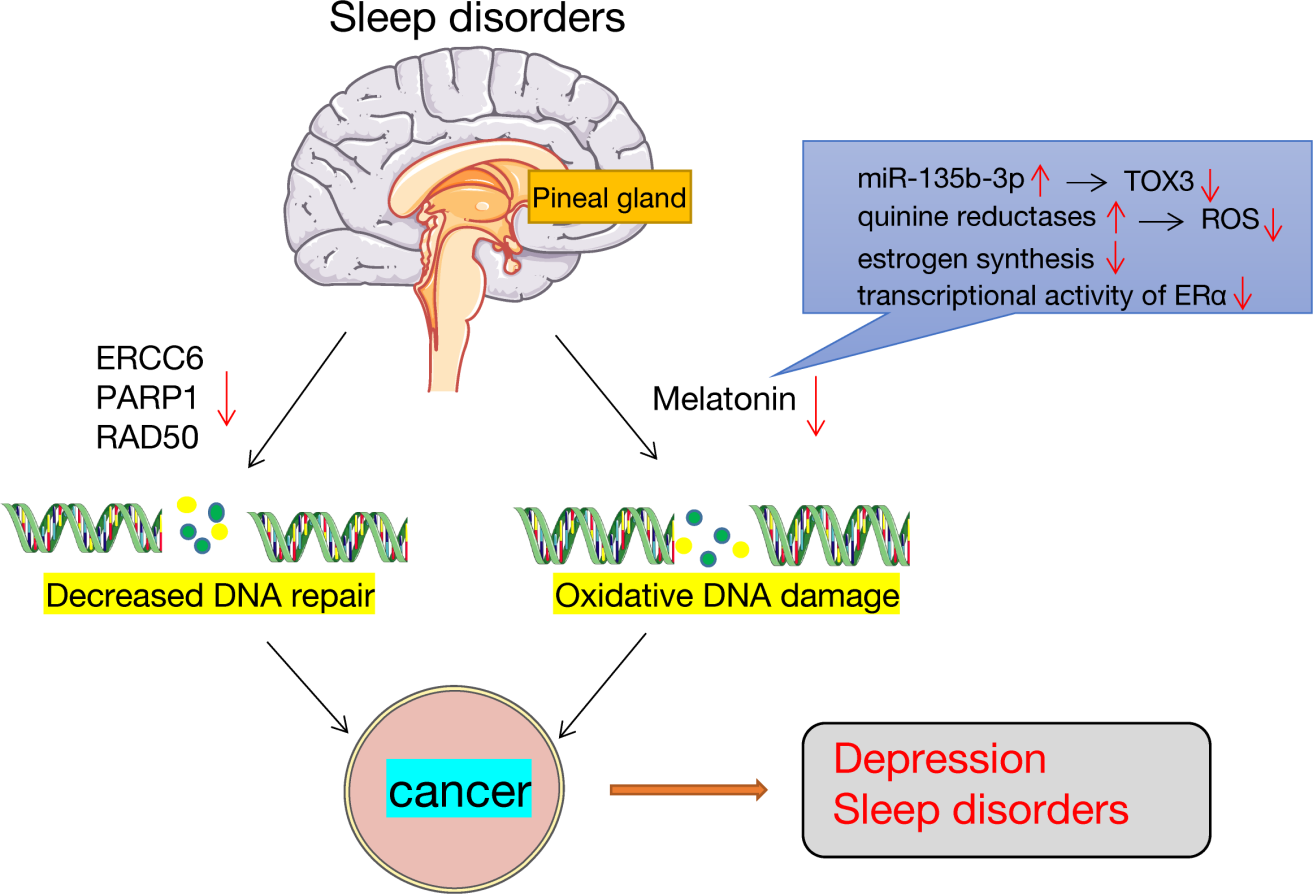
Sleep disorders, DNA damage and repair. Sleep disorders lead to the occurrence of cancers. On the one hand, sleep disorders can lower melatonin levels, which are important antioxidants that may lead to increased DNA oxidative damage. On the other hand, sleep disorders downregulate the expression of genes involved in DNA repair, such as ERCC6, PARP1 and RAD50.

Sleep disturbances affect DNA damage and repair. Sleep deprivation lowers melatonin levels, which increases oxidative damage of the DNA (125). Melatonin is a cancer suppressor and a potent estrogen inhibitor, and its release is dependent on the pineal gland and the suprachiasmatic nucleus. Melatonin acts primarily through the following: it can reduce TOX3 expression by directly activating miR-135b-3p, to thereby inhibit cancer cell migration and proliferation (126); second, melatonin affects is a strong free radical scavenger that impacts quinine reductases to reduce the oxidative damage caused by ROS in tissues (127); finally, melatonin is a strong repressor of transcriptional activity of estrogen-induced estrogen receptor-α (ERα) and inhibits ovarian estrogen production (128), both of which have regulatory roles in the HPG axis. Elevated estrogen levels are generally linked to a higher risk of developing cancer (129). In addition, sleep deprivation can suppress the expression of some genes associated with DNA repair, including RAD50, PARP1, and ERCC6, which eventually leads to the growth of tumors. Conversely, cancer-induced sleep disorders is also associated with oxidative DNA damage and repair (**Figure 3**).

Other less clear mechanisms include changes in blood glucose levels, amino acid concentrations, and pH levels. So far, the link between sleep cancer and depression is not fully elucidated, and more research is needed.

## 8 Conclusion and discussion

People with cancer often experience sleep disorders after cancer treatment, but they are often underestimated in practice. Sleep disorders symptom often overlap with cancer pain or physical fatigue and is difficult to differentiate, in addition to the accuracy of screening tools, the diversity of cancer types and treatment modalities. All this makes current research on sleep disorders in patients with cancer challenging. Based on the literature, the causes of the symptom in patients with cancer is not only related to physical pain, digestive tract reaction, fatigue, nervous system changes caused by primary cancer and treatment methods, such as surgery, chemotherapy and radiation, gender, age, primary disease, marital status, the levels of education and economic, and social support all contribute to the occurrence of sleep disorders in patients with cancer. Similarly, the development of the symptom in turn increases the incidence of cancer, affects cognition and memory, reduces quality of life, and ultimately reduces the survival rate of patients with cancer. Current research suggests that sleep disorders may be an early signal of cancer, and as sleep problems are modifiable lifestyle factors, early screening and intervention for sleep disorders may provide unique intervention targets for cancer prevention. Future research should focus on reducing risk factors for sleep disorders in patients with cancer, establishing standardized assessment tools to measure sleep disorders, providing regular monitoring, early identification, and effective management, and evaluating the long-term benefits of sleep interventions in cancer prevention and treatment. At present, drug therapy is still the main treatment for sleep disorders in patients with cancer. However, research has shown that drug therapy has many side effects and is more suitable for short-term use. Non drug treatments such as psychological intervention, cognitive-behavioral therapy, music therapy, exercise therapy, massage and relaxation therapy have fewer side effects and costs, and are beneficial in the long term, which will become the main treatment method for these symptoms in the future. Integrating sleep management into cancer treatment regimens and incorporating routine screening for sleep disorders into standard processes of cancer care can significantly improve the effectiveness of cancer treatment and the quality of life of patients with cancer, it is essential not only for personal health, but also for clinical practice and public health.

While there is compelling data on the interplay between cancer and sleep disorders involving aspects of the circadian rhythm, nervous system, endocrine system, immune system, inflammatory response, and DNA. However, prospective studies of these complications in cancer survivors have been limited, with time and ethnicity limitations, and findings have been mostly exploratory rather than explanatory. Based on the history of tumor and sleep disorders in recent 20 years, the clinical treatment of tumor-induced sleep disorders and their direct bidirectional mechanisms may be a new focus of future research. The study of intrinsic mechanisms will help to reduce the occurrence of such complications at source, which is both a challenge and an opportunity for researchers. Future research can consider utilizing information from big data platforms, combined with genomics and metabolomics, to gain a deeper understanding of the potential mechanisms of cancer and sleep disorders and develop new treatment strategies to benefit patients with cancer and improve their quality of life and survival rate.
